# PMBD: a Comprehensive Plastics Microbial Biodegradation Database

**DOI:** 10.1093/database/baz119

**Published:** 2019-11-18

**Authors:** Zhiqiang Gan, Houjin Zhang

**Affiliations:** 1 Department of Biotechnology, College of Life Science, Huazhong University of Science and Technology, Email: 279659072@qq.com; 2 Key Laboratory of Molecular Biophysics, Ministry of Education, Wuhan, Hubei, China

## Abstract

Since the invention over a hundred years ago, plastics have been used in many applications, and they are involved in every aspect of our lives. The extensive usage of plastics results in a tremendous amount of waste, which has become a severe burden on the environment. Several degradation approaches exist in nature to cope with ever-increasing plastic waste. Among these approaches, biodegradation by microorganisms has emerged as a natural way, which is favored by many environmentally conscious societies. To facilitate the study on biodegradation of plastics, we developed an online resource, Plastics Microbial Biodegradation Database (PMBD), to gather and present the information about microbial biodegradation of plastics. In this database, 949 microorganisms–plastics relationships and 79 genes involved in the biodegradation of plastics were manually collected and confirmed through literature searching. In addition, more than 8000 automatically annotated enzyme sequences, which were predicted to be involved in the plastics biodegradation, were extracted from the TrEMBL section of the UniProt database. The PMBD database is presented with a website at http://pmbd.genome-mining.cn/home. Data may be accessed through browsing or searching. Also included on the website are a sequence alignment tool and a function prediction tool.

## Introduction

Plastics are essential synthetic polymers that are widely used in different industries. The word plastic is derived from the Greek word ‘plastikos’, meaning the substance that can be molded into any shape (1). More than 50 years ago, synthetic polymers started to replace natural materials in many areas. Nowadays, plastics play an indispensable role in our everyday life (2, 3). Chemically, plastics are long hydrocarbon chain polymers with high molecular weights. Plastics are mainly derived from petrochemicals, which are further synthetically arranged to produce long-chain polymers (4, 5).

The rapid development of the global economy has led to a massive demand for plastics. The consumption of plastics increased from 1.5 million tons per year in 1950 to an incredible 265 million tons per year in 2011 (6). Fifty-six million tons were produced in 2013 alone for polyethylene terephthalate (PET). According to the U.S. Environmental Protection Agency (www.epa.gov), about 31 million tons of plastic waste were produced in 2010, which represents ~12.4% of the total Municipal Solid Waste. And out of that, only 8% of the waste was recycled (1). Due to the widespread use of plastics and the difficulties in degrading the plastics, it has become one of the most persistent pollutants in the environment (2, 7–10). A lot of plastic waste has ended up in the oceans. A new study has shown that a vast ocean plastic accumulation zone in subtropical waters between California and Hawaii contains at least 79 000 tons of ocean plastics in an area of 1.6 million km^2^ (11).

The main methods used to treat plastic waste are landfill, incineration and recycling. Each of these methods has its drawbacks. The plastics in the landfill will last for a very long time without rotting. The plastics buried in the soil makes it useless because the land becomes too soft to hold any building. The incineration of plastics releases toxic gases, which are a pollutant to the environment. And it is usually expensive to recycle plastics (12, 13). Except for these methods, the biodegradation by microorganisms has proven to be an environment-friendly way to degrade plastic waste. Although recycling is still the most preferred method, biodegradation is desirable for plastics with specific applications, such as agricultural mulch films. Significant progress has been made in related research areas. For example, the biodegradable plastics (polyesters), including polyhydroxyalkanoates (PHA), poly (butylene adipate-co-terephthalate) (PBAT), poly(ethylene furanoate) (PEF), polycaprolactone, aliphatic polyesters, polysaccharides and copolymer, or a blend of these, have been developed successfully over the last few years (14, 15). These biodegradable polyesters have also been used in many applications. For example, PBAT is a biodegradable alternative to polyethylene (PE) films, and PEF is a biodegradable alternative to PET bottles (16, 17). PHA are used as biodegradable packaging materials in the food industry or in the biomedical field as biocompatible medical implants, biodegradable sutures, skin substitutes, etc. (18). Polylactic acid (PLA) was developed as a versatile biodegradable thermoplastic that can be converted into a variety of aliphatic polyesters (1). Meanwhile, more and more microorganisms have been found to degrade plastics that were once thought resistant to biodegradation. Biodegradation of plastics by microorganisms is more widely spread in nature than we thought (7–10, 19–21). Many efforts have been made to study the enzymes involved in the biodegradation process (10, 22–33). Although there is an increasing amount of research about plastic biodegradation in the literature, there is still a lack of resources containing information on microbial degradation of plastics. Therefore, it is a meaningful task to collect the relevant information in the literature and establish a resource to display the latest data about microbial degradation of plastics.

In this study, the National Center for Biotechnology Information (NCBI) database was used to search for information about plastics biodegradation by microbes. Subsequently, useful information was extracted from the literature manually. Also, automatically annotated enzyme sequences were collected from the UniProt database (34). Based on this information, a comprehensive Plastics Microbial Biodegradation Database (PMBD) was constructed to allow users to access our data. The data consist of microorganisms and enzymes associated with plastic biodegradation, which are confirmed by the literature, and annotated sequences predicted to be related to plastic degradation. The online server is implemented in Django/MariaDB/JavaScript, and it is freely accessed through http://pmbd.genome-mining.cn/home. To the best of our knowledge, this is the first database designed for plastics biodegradation.

## Abbreviations

PE, polyethylene; PVA, polyvinyl alcohol; DMT, dimethyl terephthalate; PU, polyurethane; PET, polyethylene terephthalate; PA, phthalic acid; PHB, poly(3hydroxybutyrate); DMP, dimethyl phthalate; BBP, butyl benzyl phthalate; PCL, polycaprolactone; TA, terephthalic acid; PP, polypropylene; PLA, polyactic acid; PBA, poly (butylene adipate-co-terephthalate); PBSA, poly (butylene succinate-co-adipate); PHBH, poly(3-Hydroxybutyrate-co-3-Hydroxyhexanoate); PBS, polybutylene succinate; PES, polyethylene succinate; DBP, dibutyl phthalate; IA, isophthalic acid; PS, polystyrene; DEP, diethyl phthalate; PPA, polyphthalamide; PEA, polyethylene adipate; PCTG, sky-green; DEHP, di(2-ethyl hexyl) phthalate; PVC, polyvinyl chloride; DETP, diethyl terephthalate; PEF, poly (ethylene furanoate); PEG, polyethylene glycol.

## Materials and Methods

### Data collection

To gather information about microbial biodegradation of plastics, numerous keywords, such as ‘biodegradation’, ‘bioremediation’, ‘depolymerization’ and ‘enzyme’, were combined with plastic names to be used as the input for the search in the NCBI PubMed database. To collect the most relevant papers, the ‘title/abstract’ or other options were chosen as the search filter (Supplementary File). Both abbreviations and full names of plastics were used to maximize the search result.

The collected literature was curated manually. The connections between the microorganisms and the plastic waste were confirmed by the description of such a relationship in the literature. In particular, if a specific gene is described in the literature to be involved in the plastic biodegradation, the GenBank ID of the gene is collected. Then, the redundancy of the data, such as identical biodegradation relationships of microorganisms and plastics, was removed through manual curation. For example, if multiple papers describe the same biodegradation relationship, such a connection was only used to construct one entry in the database.

**Figure 1:**
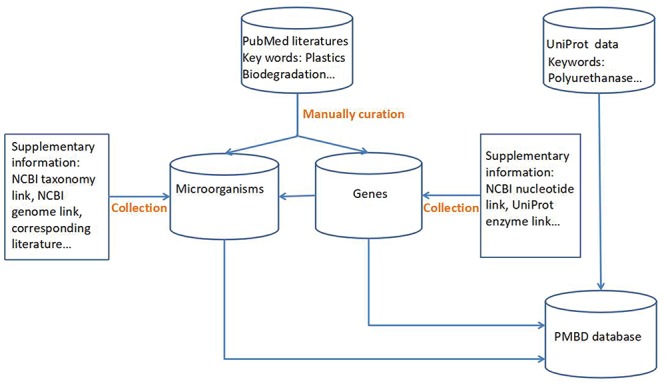
Data collection flow chart.

In addition to the data extracted from literature, more than 8000 enzymes related to plastic degradation from the UniProt database were obtained by keyword searching, such as ‘poly(3-hydroxyalkanoate) depolymerase’, ‘poly(3-hydroxybutyrate) depolymerase’, etc. These sequences are from the UniProt database TrEMBL section, which is a section containing enzyme sequences annotated automatically but not reviewed. The biodegradation functions of these enzymes remain to be verified (Figure 1).

**Figure 2:**
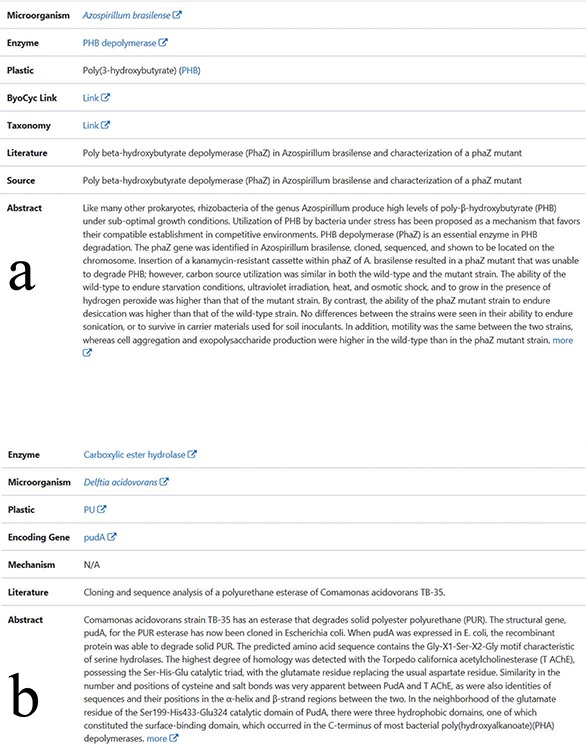
The display of microorganisms or enzymes entries. (a) The display content for each entry of manually collected biodegradation relationships between microorganisms and plastics. (b) The display content for each entry of enzymes with plastics biodegradation function.

**Figure 3:**
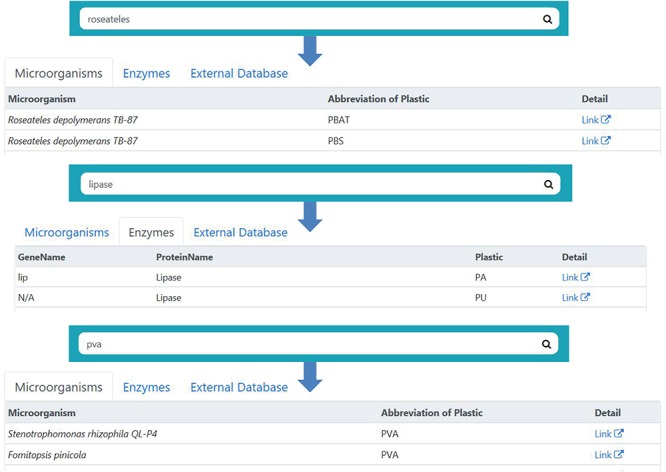
The search bar on the PMBD website. A search bar is provided for the users to access desired data. The search bar has a simple design, while the search results are grouped into microorganisms, enzymes and external database categories. The users may access the data in a specific category by pressing the corresponding button.

**Figure 4:**
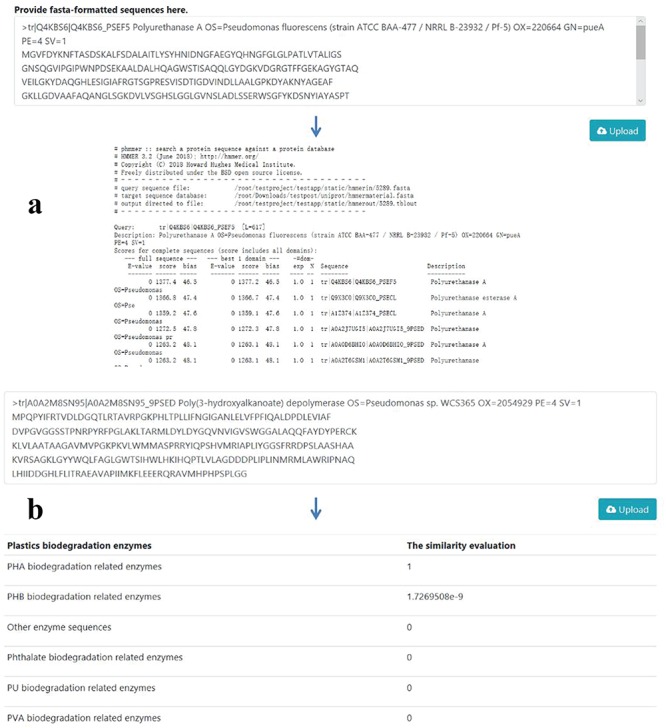
PMBD tools based on the sequences collected in the database. (a) Sequence alignment tool. Users may upload protein sequence in FASTA format. The sequences will be aligned with the sequences in the database with Hmmer. (b) Function prediction tool based on a CNN network. The potential biodegradation function of the inputted sequences will be predicted with a model trained with enzyme sequences in the database.

**Figure 5:**
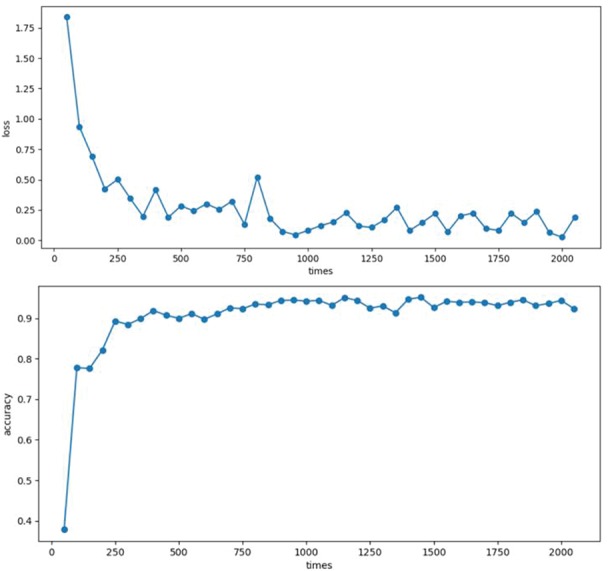
The loss and accuracy plot. The accuracy of the model increased gradually during the training process.

### Data management

The collected data consist of microorganisms and enzymes manually extracted from the literature and the automatically annotated enzyme sequences obtained from the UniProt database. For clarity, these data were organized into two categories.

The first category includes the information of the extracted microorganisms and enzymes from the literature. Each pair of microorganisms and plastics, which are involved in the same biodegradation process, was used to construct an entry. Also, in the entry are the NCBI taxonomy links for the microorganisms, the NCBI genome links for the microorganisms, the abbreviations and full names for the corresponding plastics, the related literature with their download links, abstracts and the related descriptions in the literature. For some entries, the information about the enzymes involved in the biodegradation process is also listed, if it has been described in the literature. In the entry corresponding to each enzyme extracted from literature, the UniProt link for the enzyme, its NCBI nucleotide link for the encoding gene (35) and the corresponding plastics are collected and displayed on a web page (36). In addition, relevant literature with the download links and abstracts are also listed on the same page. For some enzyme entries, the mechanisms about how the biodegradation of the plastic occurs were obtained if they are available.

The second category is stored in the section of the website called ‘Predicted’. Each entry of this category contains the UniProt ID of the enzyme, the protein name, the coding gene name, the microorganism and the corresponding biodegradable plastic.

### Website building

The MariaDB 5.5.56 was used to manage the data. The various functions of the website were implemented with Django 1.8 framework and Python 2.7. The layout of the website page, such as the style of the tables, the style of the input box and the design of the navigation bar, was set up with Bootstrap 4 package. The JavaScript 2.0 was used to complete the front-end functions, such as processing the data from the back-end, generating tables to display the data, generating the navigation bar to provide links for other pages, etc. Since JavaScript is used to implement the essential functions of these pages, browsing the pages requires the JavaScript-enabled browsers. In addition, the sequence alignment tool Hmmer 3.2 was incorporated to help users align their uploaded enzyme sequences with the sequences in our database (37). Furthermore, a sequence prediction tool based on a convolution neural network (CNN) is provided for the users to predict the potential plastics biodegradation activities of their uploaded sequences.

## Results

### Access the data

The data of PMBD could be accessed freely at its website http://pmbd.genome-mining.cn/home/. On the top of the front page, a banner is located, which contains the title and several links leading to different sections of the database. Also, on the banner is the search bar that provides quick access to the data in the database. In the middle of the front page is the rolling display of pictures of plastic waste. Underneath the display is a brief description of the rationality behind this database. Also, on the front page is the graphic representation of the database, where various links are embedded. The users can navigate to the desired web pages by clicking on the graphics.

In PMBD, the data are divided into two categories: the experimental confirmed data and predicted enzymes, which may be involved in the biodegradation process. The experimentally confirmed data can be accessed through the link ‘Confirmed’ on the front page. This link leads to the web page that contains several links. In the ‘The Microorganisms’ link, the names of the microorganisms are listed alphabetically and displayed along with the plastics names. For each microorganism, a web page is provided to display detailed information, as described in the method section. The information includes the taxonomy link in NCBI, abbreviation and the full name of plastic, biocyc link for the microorganism, download link of related literature, description of plastic–microbe relationship in this literature and the abstract of the literature (Figure 2a) (38). In the ‘The Enzymes’ link, the names of all the enzymes are displayed along with gene names and the plastics names. For each enzyme, a web page is assigned to display detailed information, including the plastic that the enzyme degrades, the microorganism where the enzyme originates from and the literature that describes the function of the gene (Figure 2b) (39). In order to show the in-depth understanding of plastic biodegradation, the mechanisms of some biodegradation processes were collected from literature and displayed at the ‘The Mechanisms’ link. Each page within this link contains the illustration of the mechanism, the names of the microorganisms, the genes, the enzymes coded by the genes and the references in which the mechanism was proposed. To help users access the mechanisms of depolymerization, the mechanisms of depolymerization were grouped and displayed in the section designated as ‘depolymerization mechanisms’. The mechanisms of further degradation are put in the section designated as ‘downstream degradation mechanisms’.

The predicted sequences of enzymes from the UniProt database are stored in the ‘Predicted’ link. The enzymes are divided into several groups based on the plastics that they may degrade. Currently, the enzymes for the degradation of PHA, PHB, PU, PVA, PET and PLA are listed. Each group is assigned with a web page displaying the protein names, the gene names, the microorganisms, the lengths of the enzyme sequences and the target plastics. In addition, the entry ID in the UniProt database is listed to provide additional information about the enzyme.

To make it easier for users to access the data that they need, a search bar is provided at the top portion of each web page throughout the site. The search function looks for the keywords in full names or the abbreviations, such as ‘Polyvinyl alcohol’ or ‘PVA’. So users can use either full names or abbreviations as the keywords to reach the same entry (Figure 3). The search results are divided into three categories, which can be accessed separately by pressing the ‘Microorganisms’, ‘Enzymes’ or ‘External Database’ buttons. In addition to the search function, the download links for all of our data, including microorganisms, genes, literature and predicted enzyme sequences are provided in the ‘Downloads’ page.

### Sequence alignment and function prediction

To analyze the similarity between the user-provided sequence and the sequences in the PMBD database, the alignment tool Hmmer is embedded in the website to provide the function of sequence alignment (Figure 4a). The default *E*-value is kept as 0.001 as the Hmmer program suggests. The alignment result displays all the similar sequences along with the scores, *E*-values and biases. To help users predicting the potential plastics biodegradation function of a protein sequence, a CNN model was trained to classify the enzyme sequences in the PMBD database. Invented initially for computer vision, CNN models have proven to be useful in processing sequential information, such as natural language processing (NLP) (40). Therefore, we use the NLP approaches in which enzyme sequences were treated as small pieces of text. A sequence classification model was constructed using CNN, and the project can be accessed through https://github.com/LaboratoryOfGenomeMining/cnnclassificationmodel.

In the training process, firstly, the enzyme sequence data, containing 1151 PHA depolymerase sequences, 1221 PHB depolymerase sequences, 205 polyurethanase sequences, 732 PVA dehydrogenase sequences and 2301 enzyme sequences related to phthalate biodegradation, were divided into the training set, the test set and the validation set. Then each sequence was turned into a matrix by one-hot coding method. The size of the matrix is 20 x *N*, in which *N* is the length of the protein sequence. Secondly, the network was built with three convolutional layers, with 16, 32 or 64 convolution kernels, respectively, to extract the information in the sequence. Then two fully connected layers divide the extracted data into six categories, which correspond to PVA, PU, PHB, PHA, Phthalate and a negative class. The network was trained for many times. Each time, the calculated loss decreased, and the classification accuracy in the test set increased (Figure 5).

Then, five models were used to test their performance in the validation set. Their validation accuracies are as follows: 84.3%, 87.2%, 86.7%, 85.2% and 83.1%. The model with the highest score (Table S1) was incorporated into the server. Also, an interface is provided for the user to upload the protein sequences to predict their functions in plastics biodegradation (Figure 4b).

**Figure 6:**
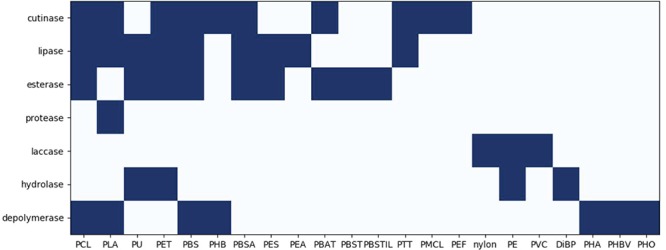
The correlation between enzymes and plastics. The dark blocks indicate interaction between enzymes and plastics.

### Data upload and database update

To improve the user experience of PMBD, two interactive features are incorporated in the website. One feature is for users to leave messages. If users find errors on the website or want to contact us, they may leave messages and their contact information on the page. This information will be recorded automatically after they upload the messages. Another feature is for users to upload data. As the literature on NCBI is continuously updated, PMBD needs to be updated periodically. If users find any information related to the plastic biodegradation that is not included in our database, they can fill in microorganisms, plastics, literature sources and their contact information on the page and upload them. We will check the user uploaded data every month and update our database accordingly. At the same time, we will search the NCBI website for new information about plastic biodegradation and update the database. These two user-interactive functions for leaving a message and uploading data can be accessed through the two links at the bottom of every page at the PMBD website.

## Discussion

To create the database, research papers were collected from PubMed through keyword searching. Then the papers were curated manually to find the experimental data supporting plastic biodegradation. The microorganisms and enzymes, which were clearly indicated by the literature to be involved in the plastic biodegradation, are incorporated into the database. Ideally, to support the claim of plastic biodegradation, multiple lines of evidence should be provided, which include loss of weight, loss of turbidity, change of surface morphology, detection of degradation products, etc. However, it is noticeable that different literature provides different sets of data to suggest plastics biodegradation. Some of the papers, especially the earlier papers, only performed turbidity assay or weight loss assay to test the plastic biodegradation. Although such an assay is considered insufficient by today’s standard, we cannot rule out the degradation activities. Also, many potential users of this database may have learned useful information from these papers. Therefore, these data were put into the PMBD database also. However, given the limited amount of evidence in some papers, we cannot guarantee that there is no false positive. We provide a search bar for the users to extract relevant information from the database. Eventually, the users will decide which information they will use.

The biodegradation of plastics involves the initial depolymerization and the further degradation of the small molecules. It is crucial to distinguish between enzymes that are involved in these two distinctive steps. However, in the current version of PMBD database, these enzymes are not organized into separate classes, which may cause confusion for the users. This shortcoming will be corrected in the updated version of this database.

**Figure 7:**
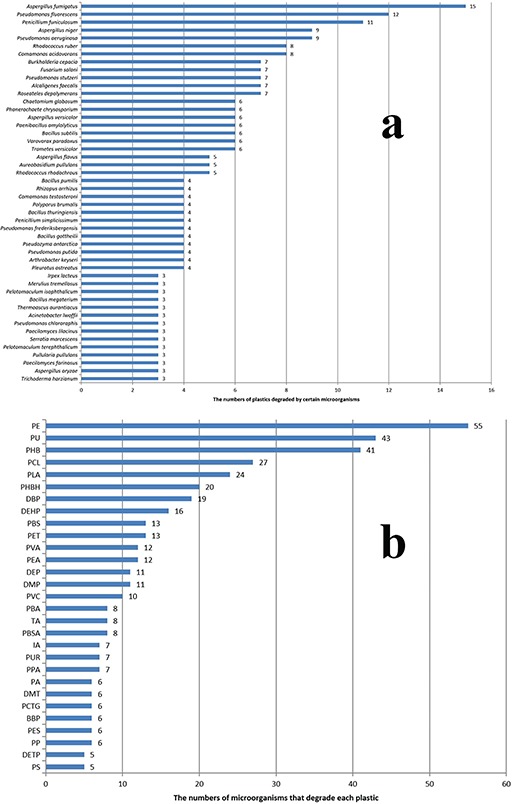
The numeric demonstration of biodegradation relationships between microorganisms and plastics. (a) The numbers of plastics that may be degraded by the listed microorganisms. (b) The numbers of biodegrading microorganisms correspond to each plastic type.

The degradation of different plastics requires a different set of enzymes (10, 39). Most of the plastics are polyesters, whose biodegradation is usually catalyzed by enzymes such as cutinases or esterases (41). Unexpectedly, also found in the database is the protease, whose target compound is PLA. In the PLA degradation process, the long polymer chain was first degraded by the depolymerase. Then, the serine proteases, such as protease K and trypsin, degrade it further into low molecular weight compounds (42–44). It has been reported that these PLA-degrading proteases come from many microorganisms, such as *Amycolatopsis*, *Saccharothrix*, *Pseudonocardia*, etc. Interestingly, it looks that these proteases are only involved in the degradation of PLA. In contrast, for other enzymes in the PMBD, they target many kinds of plastics. For example, cutinases can degrade PCL, PLA, PET, etc. And lipases can degrade PCL, PLA, PBS, etc. (Figure 6). It seems that a variety of enzymes are involved in the degradation of plastics, while the same plastic may be degraded by different enzymes. Likewise, many bacteria can degrade a broad spectrum of plastics. As shown in Figure 7a, *Aspergillus fumigatus*, *Pseudomonas fluorescens*, *Penicillium funiculosum* can degrade 10 or more types of plastics. *Rhodococcus ruber*, *Comamonas acidovorans* and *Pseudomonas aeruginosa* also show good biodegradation activities for more than five types of plastics. Plastics PE, PU and PHB can be degraded by more than 30 species of microorganisms (Figure 7b). And PLA and PCL can be degraded by more than 20 species of microorganisms. However, there is little evidence that the plastics serve as the primary nutrition sources for the microorganisms (45). It is likely that the enzymatic depolymerization of synthetic plastics is a co-metabolic process and not something enzymes evolved for.

Although many bacteria have been proven to take part in the plastics biodegradation, the study of the underlining genetic machinery lags way behind. So far, only 79 enzymes are confirmed to be responsible for the degradation. Fewer studies have been performed on the possible mechanisms of the degradation activities. Most of these mechanisms illustrate the bond cleavage step of the hydrolysis process. A few studies have demonstrated how the enzymes bind to the surfaces of the polymers and how the large polymers molecules reach the active sites of the enzymes (46, 47). The increase of the polymer-chain flexibility could increase the hydrolysis rate of PBAT by *Rhizopus oryzae* lipase and *Fusarium solani* cutinase. On the other hand, the enzymes with more accessible active sites have higher hydrolysis activities against PBAT (46). In other studies, it was shown that the fusion of cutinases and a polymer binding module could enhance the hydrolysis of the polyester poly(1,4-butylene adipate), which should be attributed to the better binding of the enzyme and the polymer (47). With plastics waste pile up in the landfill and also in the ocean, more studies should be carried out to gain detailed information about the plastics biodegradation process.

## Summary

In summary, the microorganism data, the enzyme sequences, the types of plastics and the mechanisms regarding plastics biodegradation were collected from the literature. To enrich the sequence pool, the sequences of enzymes predicted to be involved in the plastics biodegradation were retrieved from the UniProt database TrEMBL. Based on these data, a database PMBD was made, and a website was created to provide access to the database. Such a database would serve as a helpful tool to the researchers in the field of plastics microbial biodegradation.

*Conflict of interest*. None declared

## Supplementary Material

Table_S1_old_baz119

Supplement_file_baz119

## Notes

**Database URL:** http://pmbd.genome-mining.cn/home/
