# Supplementary material for: PMBD: a Comprehensive Plastics Microbial Biodegradation Database
Source: Database (Oxford). 2019 Nov 18;2019:baz119. doi: 10.1093/database/baz119 (PMC6859810; doi:10.1093/database/baz119)
Supplement: Table_S1_old_baz119 [file table_s1_old_baz119.docx]

Table S1. The accuracy of the highest-scored model in predicting different classes of enzymes

| Class | Accuracy |
| --- | --- |
| PVA biodegradation related enzymes | 88.5% |
| PU biodegradation related enzymes | 100% |
| PHA biodegradation related enzymes | 87.6% |
| PHB biodegradation related enzymes | 95.1% |
| Phthalate biodegradation related enzymes | 94.3% |
| The enzymes without the above functions | 85.6% |
