# Supplementary material for: PMBD: a Comprehensive Plastics Microbial Biodegradation Database
Source: Database (Oxford). 2019 Nov 18;2019:baz119. doi: 10.1093/database/baz119 (PMC6859810; doi:10.1093/database/baz119)
Supplement: Supplement_file_baz119 [file supplement_file_baz119.pdf]

Supplementary File

Searching keywords	Retrieved literatures	Matched literatures
(plastics[Title/Abstract]) and biodegradation[Title/Abstract]	126	30
(polymer[Title/Abstract]) and biodegradation[Title/Abstract]	769	22
(phthalate[Title/Abstract]) and biodegradation[Title/Abstract]	232	47
(PE[Title/Abstract]) and biodegradation[Title/Abstract]	30	7
(PU[Title/Abstract]) and biodegradation[Title/Abstract]	52	4
(PLA[Title/Abstract]) and biodegradation[Title/Abstract]	122	7
(PVA[Title/Abstract]) and biodegradation[Title/Abstract]	83	16
(PVC[Title/Abstract]) and biodegradation[Title/Abstract]	28	11
(PHB[Title/Abstract]) and biodegradation[Title/Abstract]	58	7
(PHA[Title/Abstract]) and biodegradation[Title/Abstract]	46	4
(PET[Title/Abstract]) and biodegradation[Title/Abstract]	28	5
(PBS[Title/Abstract]) and biodegradation[Title/Abstract]	96	1
(PBST[Title/Abstract]) and biodegradation[Title/Abstract]	0	0
(PBSA[Title/Abstract]) and biodegradation[Title/Abstract]	10	2
(PBA[Title/Abstract]) and biodegradation[Title/Abstract]	15	0
(PBAT[Title/Abstract]) and biodegradation[Title/Abstract]	11	3
(PEF[Title/Abstract]) and biodegradation[Title/Abstract]	2	2
(PCL[Title/Abstract]) and biodegradation[Title/Abstract]	147	8
(PES[Title/Abstract]) and biodegradation[Title/Abstract]	11	1
(PEA[Title/Abstract]) and biodegradation[Title/Abstract]	22	0
(PS[Title/Abstract]) and biodegradation[Title/Abstract]	74	2
(PP[Title/Abstract]) and biodegradation[Title/Abstract]	37	3
(Polyethylene[Title/Abstract]) and biodegradation[Title/Abstract]	243	28
(Polyurethane[Title/Abstract]) and biodegradation[Title/Abstract]	193	7
(Polylactic acid[Title/Abstract]) and biodegradation[Title/Abstract]	50	3
(Polyvinyl alcohol[Title/Abstract]) and biodegradation[Title/Abstract]	69	18
(Polyvinyl chloride[Title/Abstract]) and biodegradation[Title/Abstract]	18	9
(Poly(3-hydroxybutyrate)[Title/Abstract]) and biodegradation[Title/Abstract]	0	0
(Poly(3-hydroxyalkanoates)[Title/Abstract]) and biodegradation[Title/Abstract]	0	0
(Polyethylene terephthalate[Title/Abstract]) and biodegradation[Title/Abstract]	13	3
(Polybutylene succinate[Title/Abstract]) and biodegradation[Title/Abstract]	1	0
(Poly(butylene succinate-co-terephthalate)[Title/Abstract]) and biodegradation[Title/Abstract]	0	0
(Poly(butylene succinate-co-adipate)[Title/Abstract]) and biodegradation[Title/Abstract]	0	0
(Polybutylene adipate[Title/Abstract]) and biodegradation[Title/Abstract]	0	0
(Poly(butylene adipate-co-terephthalate)[Title/Abstract]) and biodegradation[Title/Abstract]	1	0
(Poly(ethylene furanoate)[Title/Abstract]) and biodegradation[Title/Abstract]	0	0
(Polycaprolactone[Title/Abstract]) and biodegradation[Title/Abstract]	84	4
(Polyethylene succinate[Title/Abstract]) and biodegradation[Title/Abstract]	2	1
(Polyethylene adipate[Title/Abstract]) and biodegradation[Title/Abstract]	3	0
(Polystyrene[Title/Abstract]) and biodegradation[Title/Abstract]	58	8
(Polypropylene[Title/Abstract]) and biodegradation[Title/Abstract]	53	5
(plastics[Title/Abstract]) and hydrolysis[Title/Abstract]	91	5
(polymer[Title/Abstract]) and hydrolysis[Title/Abstract]	2451	7
(phthalate[Title/Abstract]) and hydrolysis[Title/Abstract]	186	5
(PE[Title/Abstract]) and hydrolysis[Title/Abstract]	330	1
(PU[Title/Abstract]) and hydrolysis[Title/Abstract]	84	1
(PLA[Title/Abstract]) and hydrolysis[Title/Abstract]	388	2
(PVA[Title/Abstract]) and hydrolysis[Title/Abstract]	119	0
(PVC[Title/Abstract]) and hydrolysis[Title/Abstract]	39	0
(PHB[Title/Abstract]) and hydrolysis[Title/Abstract]	105	6
(PHA[Title/Abstract]) and hydrolysis[Title/Abstract]	128	4
(PET[Title/Abstract]) and hydrolysis[Title/Abstract]	364	4
(PBS[Title/Abstract]) and hydrolysis[Title/Abstract]	191	1
(PBST[Title/Abstract]) and hydrolysis[Title/Abstract]	4	0
(PBSA[Title/Abstract]) and hydrolysis[Title/Abstract]	15	0
(PBA[Title/Abstract]) and hydrolysis[Title/Abstract]	26	0
(PBAT[Title/Abstract]) and hydrolysis[Title/Abstract]	8	4
(PEF[Title/Abstract]) and hydrolysis[Title/Abstract]	8	0
(PCL[Title/Abstract]) and hydrolysis[Title/Abstract]	130	2
(PES[Title/Abstract]) and hydrolysis[Title/Abstract]	32	1
(PEA[Title/Abstract]) and hydrolysis[Title/Abstract]	244	0
(PS[Title/Abstract]) and hydrolysis[Title/Abstract]	521	1
(PP[Title/Abstract]) and hydrolysis[Title/Abstract]	262	0
(Polyethylene[Title/Abstract]) and hydrolysis[Title/Abstract]	448	5
(Polyurethane[Title/Abstract]) and hydrolysis[Title/Abstract]	136	2
(Polylactic acid[Title/Abstract]) and hydrolysis[Title/Abstract]	47	1
(Polyvinyl alcohol[Title/Abstract]) and hydrolysis[Title/Abstract]	71	1
(Polyvinyl chloride[Title/Abstract]) and hydrolysis[Title/Abstract]	24	1
(Poly(3-hydroxybutyrate)[Title/Abstract]) and hydrolysis[Title/Abstract]	0	0
(Poly(3-hydroxyalkanoates)[Title/Abstract]) and hydrolysis[Title/Abstract]	0	0
(Polyethylene terephthalate[Title/Abstract]) and hydrolysis[Title/Abstract]	43	5
(Polybutylene succinate[Title/Abstract]) and hydrolysis[Title/Abstract]	4	0
(Poly(butylene succinate-co-terephthalate)[Title/Abstract]) and hydrolysis[Title/Abstract]	0	0
(Poly(butylene succinate-co-adipate)[Title/Abstract]) and hydrolysis[Title/Abstract]	4	0
(Polybutylene adipate[Title/Abstract]) and hydrolysis[Title/Abstract]	1	0
(Poly(butylene adipate-co-terephthalate)[Title/Abstract]) and hydrolysis[Title/Abstract]	0	0
(Poly(ethylene furanoate)[Title/Abstract]) and hydrolysis[Title/Abstract]	0	0
(Polycaprolactone[Title/Abstract]) and hydrolysis[Title/Abstract]	61	4
(Polyethylene succinate[Title/Abstract]) and hydrolysis[Title/Abstract]	2	1
(Polyethylene adipate[Title/Abstract]) and hydrolysis[Title/Abstract]	5	0
(Polystyrene[Title/Abstract]) and hydrolysis[Title/Abstract]	206	1
(Polypropylene[Title/Abstract]) and hydrolysis[Title/Abstract]	66	0
(plastics[Title/Abstract]) and depolymerization[Title/Abstract]	27	1
(polymer[Title/Abstract]) and depolymerization[Title/Abstract]	425	2
(phthalate[Title/Abstract]) and depolymerization[Title/Abstract]	3	0
(PE[Title/Abstract]) and depolymerization[Title/Abstract]	8	1
(PU[Title/Abstract]) and depolymerization[Title/Abstract]	1	0
(PLA[Title/Abstract]) and depolymerization[Title/Abstract]	11	1
(PVA[Title/Abstract]) and depolymerization[Title/Abstract]	5	1
(PVC[Title/Abstract]) and depolymerization[Title/Abstract]	1	0
(PHB[Title/Abstract]) and depolymerization[Title/Abstract]	17	0
(PHA[Title/Abstract]) and depolymerization[Title/Abstract]	27	1
(PET[Title/Abstract]) and depolymerization[Title/Abstract]	16	0
(PBS[Title/Abstract]) and depolymerization[Title/Abstract]	9	0
(PBST[Title/Abstract]) and depolymerization[Title/Abstract]	0	0
(PBSA[Title/Abstract]) and depolymerization[Title/Abstract]	2	0
(PBA[Title/Abstract]) and depolymerization[Title/Abstract]	0	0
(PBAT[Title/Abstract]) and depolymerization[Title/Abstract]	0	0
(PEF[Title/Abstract]) and depolymerization[Title/Abstract]	1	0
(PCL[Title/Abstract]) and depolymerization[Title/Abstract]	10	0
(PES[Title/Abstract]) and depolymerization[Title/Abstract]	0	0
(PEA[Title/Abstract]) and depolymerization[Title/Abstract]	5	0
(PS[Title/Abstract]) and depolymerization[Title/Abstract]	22	0
(PP[Title/Abstract]) and depolymerization[Title/Abstract]	13	3
(Polyethylene[Title/Abstract]) and depolymerization[Title/Abstract]	30	0
(Polyurethane[Title/Abstract]) and depolymerization[Title/Abstract]	6	0
(Polylactic acid[Title/Abstract]) and depolymerization[Title/Abstract]	6	1
(Polyvinyl alcohol[Title/Abstract]) and depolymerization[Title/Abstract]	4	1
(Polyvinyl chloride[Title/Abstract]) and depolymerization[Title/Abstract]	0	0
(Poly(3-hydroxybutyrate)[Title/Abstract]) and depolymerization[Title/Abstract]	0	0
(Poly(3-hydroxyalkanoates)[Title/Abstract]) and depolymerization[Title/Abstract]	0	0
(Polyethylene terephthalate[Title/Abstract]) and depolymerization[Title/Abstract]	5	0
(Polybutylene succinate[Title/Abstract]) and depolymerization[Title/Abstract]	0	0
(Poly(butylene succinate-co-terephthalate)[Title/Abstract]) and depolymerization[Title/Abstract]	0	0
(Poly(butylene succinate-co-adipate)[Title/Abstract]) and depolymerization[Title/Abstract]	0	0
(Polybutylene adipate[Title/Abstract]) and depolymerization[Title/Abstract]	1	0
(Poly(butylene adipate-co-terephthalate)[Title/Abstract]) and depolymerization[Title/Abstract]	0	0

(Poly(ethylene furanoate)[Title/Abstract]) and depolymerization[Title/Abstract]	0	0
(Polycaprolactone[Title/Abstract]) and depolymerization[Title/Abstract]	9	0
(Polyethylene succinate[Title/Abstract]) and depolymerization[Title/Abstract]	0	0
(Polyethylene adipate[Title/Abstract]) and depolymerization[Title/Abstract]	1	0
(Polystyrene[Title/Abstract]) and depolymerization[Title/Abstract]	19	0
(Polypropylene[Title/Abstract]) and depolymerization[Title/Abstract]	5	1
(plastics[Title/Abstract]) and bioremediation[Title/Abstract]	26	9
(polymer[Title/Abstract]) and bioremediation[Title/Abstract]	75	3
(phthalate[Title/Abstract]) and bioremediation[Title/Abstract]	52	25
(PE[Title/Abstract]) and bioremediation[Title/Abstract]	8	0
(PU[Title/Abstract]) and bioremediation[Title/Abstract]	9	1
(PLA[Title/Abstract]) and bioremediation[Title/Abstract]	2	1
(PVA[Title/Abstract]) and bioremediation[Title/Abstract]	15	1
(PVC[Title/Abstract]) and bioremediation[Title/Abstract]	9	8
(PHB[Title/Abstract]) and bioremediation[Title/Abstract]	10	1
(PHA[Title/Abstract]) and bioremediation[Title/Abstract]	10	0
(PET[Title/Abstract]) and bioremediation[Title/Abstract]	12	2
(PBS[Title/Abstract]) and bioremediation[Title/Abstract]	6	1
(PBST[Title/Abstract]) and bioremediation[Title/Abstract]	1	1
(PBSA[Title/Abstract]) and bioremediation[Title/Abstract]	1	1
(PBA[Title/Abstract]) and bioremediation[Title/Abstract]	10	0
(PBAT[Title/Abstract]) and bioremediation[Title/Abstract]	1	1
(PEF[Title/Abstract]) and bioremediation[Title/Abstract]	0	0
(PCL[Title/Abstract]) and bioremediation[Title/Abstract]	2	1
(PES[Title/Abstract]) and bioremediation[Title/Abstract]	6	2
(PEA[Title/Abstract]) and bioremediation[Title/Abstract]	11	0
(PS[Title/Abstract]) and bioremediation[Title/Abstract]	25	0
(PP[Title/Abstract]) and bioremediation[Title/Abstract]	12	0
(Polyethylene[Title/Abstract]) and bioremediation[Title/Abstract]	30	6
(Polyurethane[Title/Abstract]) and bioremediation[Title/Abstract]	21	4
(Polylactic acid[Title/Abstract]) and bioremediation[Title/Abstract]	1	0
(Polyvinyl alcohol[Title/Abstract]) and bioremediation[Title/Abstract]	17	2
(Polyvinyl chloride[Title/Abstract]) and bioremediation[Title/Abstract]	5	5
(Poly(3-hydroxybutyrate)[Title/Abstract]) and bioremediation[Title/Abstract]	0	0
(Poly(3-hydroxyalkanoates)[Title/Abstract]) and bioremediation[Title/Abstract]	0	0
(Polyethylene terephthalate[Title/Abstract]) and bioremediation[Title/Abstract]	2	2
(Polybutylene succinate[Title/Abstract]) and bioremediation[Title/Abstract]	0	0
(Poly(butylene succinate-co-terephthalate)[Title/Abstract]) and bioremediation[Title/Abstract]	0	0
(Poly(butylene succinate-co-adipate)[Title/Abstract]) and bioremediation[Title/Abstract]	0	0
(Polybutylene adipate[Title/Abstract]) and bioremediation[Title/Abstract]	0	0
(Poly(butylene adipate-co-terephthalate)[Title/Abstract]) and bioremediation[Title/Abstract]	0	0
(Poly(ethylene furanoate)[Title/Abstract]) and bioremediation[Title/Abstract]	0	0
(Polycaprolactone[Title/Abstract]) and bioremediation[Title/Abstract]	2	0
(Polyethylene succinate[Title/Abstract]) and bioremediation[Title/Abstract]	5	2
(Polyethylene adipate[Title/Abstract]) and bioremediation[Title/Abstract]	0	0
(Polystyrene[Title/Abstract]) and bioremediation[Title/Abstract]	11	2
(Polypropylene[Title/Abstract]) and bioremediation[Title/Abstract]	7	1
(enzyme[Title/Abstract]) AND plastics[Title/Abstract]	164	9
(enzyme[Title/Abstract]) AND polymer[Title/Abstract]	4704	10
(enzyme[Title/Abstract]) AND phthalate[Title/Abstract]	430	8
(enzyme[Title/Abstract]) AND PE[Title/Abstract]	1384	0
(enzyme[Title/Abstract]) AND PU[Title/Abstract]	197	0
(enzyme[Title/Abstract]) AND PLA[Title/Abstract]	846	4
(enzyme[Title/Abstract]) AND PVA[Title/Abstract]	270	9
(enzyme[Title/Abstract]) AND PVC[Title/Abstract]	110	1
(enzyme[Title/Abstract]) AND PHB[Title/Abstract]	276	16
(enzyme[Title/Abstract]) AND PHA[Title/Abstract]	853	6
(enzyme[Title/Abstract]) AND PET[Title/Abstract]	1800	7
(enzyme[Title/Abstract]) AND PBS[Title/Abstract]	1205	0
(enzyme[Title/Abstract]) AND PBST[Title/Abstract]	22	0
(enzyme[Title/Abstract]) AND PBSA[Title/Abstract]	117	0
(enzyme[Title/Abstract]) AND PBA[Title/Abstract]	125	0
(enzyme[Title/Abstract]) AND PBAT[Title/Abstract]	7	5
(enzyme[Title/Abstract]) AND PEF[Title/Abstract]	106	2
(enzyme[Title/Abstract]) AND PCL[Title/Abstract]	195	7
(enzyme[Title/Abstract]) AND PES[Title/Abstract]	150	1
(enzyme[Title/Abstract]) AND PEA[Title/Abstract]	1476	0
(enzyme[Title/Abstract]) AND PS[Title/Abstract]	1881	0
(enzyme[Title/Abstract]) AND PP[Title/Abstract]	1441	0
(enzyme[Title/Abstract]) AND Polyethylene[Title/Abstract]	2125	13
(enzyme[Title/Abstract]) AND Polyurethane[Title/Abstract]	263	9
(enzyme[Title/Abstract]) AND Polylactic acid[Title/Abstract]	43	1
(enzyme[Title/Abstract]) AND Polyvinyl alcohol[Title/Abstract]	230	9
(enzyme[Title/Abstract]) AND Polyvinyl chloride[Title/Abstract]	77	1
(enzyme[Title/Abstract]) AND Poly(3-hydroxybutyrate)[Title/Abstract]	0	0
(enzyme[Title/Abstract]) AND Poly(3-hydroxyalkanoates)[Title/Abstract]	0	0
(enzyme[Title/Abstract]) AND Polyethylene terephthalate[Title/Abstract]	61	5
(enzyme[Title/Abstract]) AND Polybutylene succinate[Title/Abstract]	6	0
(enzyme[Title/Abstract]) AND Poly(butylene succinate-co-terephthalate)[Title/Abstract]	0	0
(enzyme[Title/Abstract]) AND Poly(butylene succinate-co-adipate)[Title/Abstract]	0	0
(enzyme[Title/Abstract]) AND Polybutylene adipate[Title/Abstract]	1	0
(enzyme[Title/Abstract]) AND Poly(butylene adipate-co-terephthalate)[Title]	0	0
(enzyme[Title/Abstract]) AND Poly(ethylene furanoate)[Title/Abstract]	0	0
(enzyme[Title/Abstract]) AND Polycaprolactone[Title/Abstract]	78	6
(enzyme[Title/Abstract]) AND Polyethylene succinate[Title/Abstract]	1	0
(enzyme[Title/Abstract]) AND Polyethylene adipate[Title/Abstract]	6	0
(enzyme[Title/Abstract]) AND Polystyrene[Title/Abstract]	1317	0
(enzyme[Title/Abstract]) AND Polypropylene[Title/Abstract]	162	0
